# Cocaine Enhances HIV-1 Infectivity in Monocyte Derived Dendritic Cells by Suppressing microRNA-155

**DOI:** 10.1371/journal.pone.0083682

**Published:** 2013-12-31

**Authors:** Jessica Napuri, Sudheesh Pilakka-Kanthikeel, Andrea Raymond, Marisela Agudelo, Adriana Yndart-Arias, Shailendra K. Saxena, Madhavan Nair

**Affiliations:** 1 Department of Immunology, Herbert Wertheim College of Medicine, Florida International University, Miami, Florida, United States of America; 2 Institute of NeuroImmune Pharmacology, Herbert Wertheim College of Medicine, Florida International University, Miami, Florida, United States of America; 3 Center for Personalized Nanomedicine, Herbert Wertheim College of Medicine, Florida International University, Miami, Florida, United States of America; 4 CSIR-Centre for Cellular & Molecular Biology, Hyderabad, India; Temple University School of Medicine, United States of America

## Abstract

Cocaine and other drugs of abuse increase HIV-induced immunopathogenesis; and neurobiological mechanisms of cocaine addiction implicate a key role for microRNAs (miRNAs), single-stranded non-coding RNAs that regulate gene expression and defend against viruses. In fact, HIV defends against miRNAs by actively suppressing the expression of polycistronic miRNA cluster miRNA-17/92, which encodes miRNAs including miR-20a. IFN-g production by natural killer cells is regulated by miR-155 and this miRNA is also critical to dendritic cell (DC) maturation. However, the impact of cocaine on miR-155 expression and subsequent HIV replication is unknown. We examined the impact of cocaine on two miRNAs, miR-20a and miR-155, which are integral to HIV replication, and immune activation. Using miRNA isolation and analysis, RNA interference, quantitative real time PCR, and reporter assays we explored the effects of cocaine on miR-155 and miR-20 in the context of HIV infection. Here we demonstrate using monocyte-derived dendritic cells (MDCCs) that cocaine significantly inhibited miR-155 and miR-20a expression in a dose dependent manner. Cocaine and HIV synergized to lower miR-155 and miR-20a in MDDCs by 90%. Cocaine treatment elevated LTR-mediated transcription and PU.1 levels in MDCCs. But in context of HIV infection, PU.1 was reduced in MDDCs regardless of cocaine presence. Cocaine increased DC-SIGN and and decreased CD83 expression in MDDC, respectively. Overall, we show that cocaine inhibited miR-155 and prevented maturation of MDDCs; potentially, resulting in increased susceptibility to HIV-1. Our findings could lead to the development of novel miRNA-based therapeutic strategies targeting HIV infected cocaine abusers.

## Introduction

Despite thirty years of research efforts, over 34 million people are living with Human Immunodeficiency Virus (HIV) today. In the last decade, we observed an entangled epidemic of HIV and drugs of abuse. Cocaine, one of the most widely abused drugs in the United States, has been shown to exacerbate HIV disease progression and HIV-associated neurocognitive disorders (HAND) [1]–[3]. Previously we reported that cocaine promotes HIV-1 replication through the induction of Dendritic Cell-Specific Intercellular adhesion molecule-3-Grabbing Non-integrin (DC-SIGN) expression [4]. However, the mechanism of cocaine-induced DC-SIGN modulation and HIV infectivity is not clear.

MicroRNAs (miRNA), small non-coding, single stranded RNA molecules of 19–25 nucleotides, have recently emerged as a major class of gene expression modulators [5]. miRNAs regulate gene expression by targeting the 3′-UTR (untranslated regions) of specific mRNAs for degradation or translational repression, thereby controlling protein production [6] and regulating cellular pathways. It has been shown that certain miRNA negatively regulate HIV-1 replication [7]–[9]. In fact, altered miRNA expression in HIV-infected subjects has been shown to contribute to HIV-1 latency [10]–[12]. HIV-1 actively suppresses the polycistronic miRNA cluster miR-17/92 [10], which encodes miR-17-(5p/3p), miR-18, miR-19a, miR20a, miR19b-1 and miR-92-1 [13], facilitating efficient viral replication [10]. Further studies have shown that over expression of miR-155 down-regulates DC-SIGN [14], which plays a major role in HIV infection [15] independent of CD4 and/or co-receptors [16]–[18]. Since, previous studies have shown that HIV actively suppresses the polycistronic cluster, encoding miR-20a, and miR-155, we chose to examine these two miRNAs in the context of HIV infection and drug abuse [10], [19], [20]. It is important to point out that the effects of cocaine on the expression of miR-155 and -20a have not been clearly investigated yet. Here, we demonstrate that cocaine modulates miR–155 and -20a in the context of HIV infection.

PU.1, a transcription factor [21], is required for the generation and maturation of most myeloid lineages such as macrophages, neutrophils and dendritic cells [22], [23]. PU.1, a direct transcription factor of DC-SIGN and a target of miR-155, regulates DC-SIGN in a reciprocal fashion [24], [25]. Additionally, PU.1 has been shown to regulate HIV-1 transcription by physically interacting with the NF-κB subunits within the LTR [26]. Given the impact of miR-155 on PU.1 and subsequently on DC-SIGN [21] we hypothesize that cocaine suppresses miR-155 and 20a thereby increasing DC-SIGN expression and promoting HIV-1 replication. Our results show that cocaine down- regulates miR-155 and miR-20a and modulates DC-SIGN, DC maturation, and HIV infectivity.

## Materials and Methods

### Cell culture

MDDC were differentiated from peripheral blood mononuclear cells (PBMC) as described previously [27]–[31]. Briefly, PBMC separated on a density gradient were incubated at 37°C in RPMI-1640 media containing 10% fetal bovine serum. Non-adherent cells were removed after 1 hr, and adherent cells were cultured for 6 days in media containing 100 U/ml of recombinant human GM-CSF and IL-4 (R & D systems, USA). Media were changed and cytokines were replenished every 48 hrs. At day 6, MDDCs were removed by gently splashing the surface. DC maturation was induced by culturing with the same cytokine mixtures in the presence of LPS (100 ng/ml) for additional 2 days.

### MicroRNA Analysis

MDDCs were treated with cocaine at (0.1 µM, 1 µM, 10 µM) for 15 hrs and 24 hrs. Total RNA was isolated using the mir-Vana miRNA Isolation (Ambion, USA), in accordance with manufacturer's protocol. 2 ng of each RNA sample were used for quantitative real-time RT-PCR (qRT-PCR), performed using the TaqMan MicroRNA Reverse Transcription Kit, PCR Master Mix, and specific primers (Applied Biosystems, USA). Primer sequences were; miR-155 - (UUAAUGCUAAUCGUGAUAGGGGU) and miR-20a - (UAAAGUGCUUAUAGUGCAGGUAG). All reactions were analyzed using Step One Real-Time PCR System (Applied Biosystems). miRNA levels were normalized to U6 snRNA control and the cycle threshold (Ct) values were calculated as previously described [32].

### Infection of MDDCs with HIV-1_BAL_

MDDCs were infected with HIV-1_BAL_, (NIH AIDS Research and Reference Reagent Program; catalog no. 510) at a concentration of HIV-1 p24 20 ng/10^6^cells for 2 hr as previously described [31], followed by washing to remove unbound virus. HIV-1 infected and uninfected MDDCs were cultured in the presence/absence of cocaine (1 µM) for 7 days. The culture supernatants were quantitated for p24 antigen.

### miR-155 transfection in MDCCs

MDDCs were transfected with a miR-155 mimic or anti-miR-155 using a standardized siRNA transfection protocol (Santa Cruz Biotechnology) at a final concentration of 75 nM. Following transfection, MDDCs were treated with cocaine (1 µM) and/or infected withHIV-1.

### Real Time RT-PCR Analysis

miR-155 mimic/anti-miR-155 transfected MDDCs were treated with or without cocaine and/or HIV-1. After respective treatments, cells were harvested and RNA was isolated using RNAeasy mini kit (Qiagen, Germany). The cDNA was synthesized using the high-capacity reverse transcriptase cDNA kit to perform qRT-PCR by Taqman gene expression assay using specific primers for PU.1 and (LTR)-R/U5. (primers for (LTR)-R/U5: 5′-primer, 5′-TCTCTCTGGTTAGACCAGATCTG; 3′-primer, 5′-ACTGCTAGAGATTTTCCACACTG. Values were normalized against GAPDH.

### Cytotoxicity

XTT assay (ATCC.org) was performed on MDDCs according to manufacturer's instructions. Briefly, 200 µl of cell suspension and 100 µl of XTT-PMS was plated per well in a 96 well plate and incubated for 4 hours. The cytotoxicity was measured by ELISA reader. The absorbance was measured at 450 nm with λ correction at 595 nm and correlated with control values [33]. The results are expressed as percentage of live cells.

### Plasmids, transfections, and reporter assays

The following plasmids were obtained through the NIH AIDS Reagent Program, Division of AIDS, NIAID, NIH: pHIV-CAT(cat#2619) from Dr. Gary Nabel and Dr. Neil Perkins, pSVTat (cat#294) from Dr. Alan Frankel, pNL4-3-Luc.R^-^E^-^(cat#3418) Dr. Nathaniel Landau, Aaron Diamond AIDS Research Center, The Rockefeller University, pHEF-VSV-G from Dr. Lung-Ji Chang. Amphoteric HIV pseudoviruses containing the firefly luciferase gene were produced by transfecting 293T cells with pHEF-VSV-G (10 µg) and pNL4-3.Luc.R-E- (10 µg) in 90 mm culture dishes. Briefly, plasmid DNA was diluted in Opti-MEM serum free media (Invitrogen). Lipofectamine LTX & PLUS reagent (Invitrogen) diluted in equal volume and added to diluted DNA at a 1∶1 ratio. Mixture was incubated for 5 minutes to allow formation of DNA-liposome complexes. Culture supernatants containing the pseudoviruses were collected 72 hours post-transfection and clarified by centrifugation at 400 g. Pseudoviruses were competent for single round of replication. Luciferase production measured as described below. HEK293T cells were seeded at 5×10^5^ cells in a 6-well culture dish, transfected (using Lipofectamine LTX) with pLTR-CAT (8 µg) alone or in combination with pSVTat (0.8 µg) in the presence of cocaine. Cocaine was added to cells 24 hours post transfection, and cultured for an additional 48 hours. Cells were harvested 72 hours post-transfection, lysed, and CAT concentration was measured via ELISA using Chloramphenicol acetyl-transferase (CAT) kit (Roche). Protocols performed according to manufacturer's instructions. Absorbance was measured at 405 nm (Synergy HT, BioHIT). Results were normalized to total protein concentration of samples as measured by BioRad Protein Assay (BioRad Laboratories).

### Luciferase Assay

Luciferase concentration in whole cell lysates from cells infected with HIV-luciferase pseudovirus (20 ng) in presence/absence of cocaine was determined using a Luciferase assay system (Promega), according to manufacturer's instructions (Technical Bulletin #TB281). Briefly, cells infected with the HIV-luciferase pseudovirus (20 ng) were harvested three days post infection (dpi), washed with PBS and lysed. Lysates were pre-cleared by centrifugation at 12,000 g for 2 minutes at 4°C. 100 µl of luciferase assay reagent were dispensed in a 96 well microplate (Costar), 20 µl of cell lysate added to each well, mixed by pipetting, and read on a luminometer (Synergy HT, BioHIT).

### Flow cytometry

Following respective treatments (transfection/cocaine) MDDCs and Mature DCs (MDCs) were harvested, washed with FACS buffer and incubated with Fc blocking reagent for 10 min at 4°C. Cells were stained for 30 min in the dark at RT with CD83 PE (BD Pharmingen) and DC-SIGN FITC (eBiosciences) or with appropriate isotype controls. Cells were acquired using FACS Calibur with collection of 100,000 cells and analyzed using Flowjo software.

### Statistical analysis

Results are presented as mean ± SE of three independent experiments. Differences between treated and untreated cultures were compared by Student's t-Test. Comparisons of more than two groups were made using analysis of variance (ANOVA) (Kruskal-Wallis). Data were analyzed using Graphpad Prism version 5.0 software and statistical significance considered when *p-value*≤0.05.

## Results

### Cocaine Down-regulates miR-155 and 20a Expression in MDDCs

**Figure 1:**
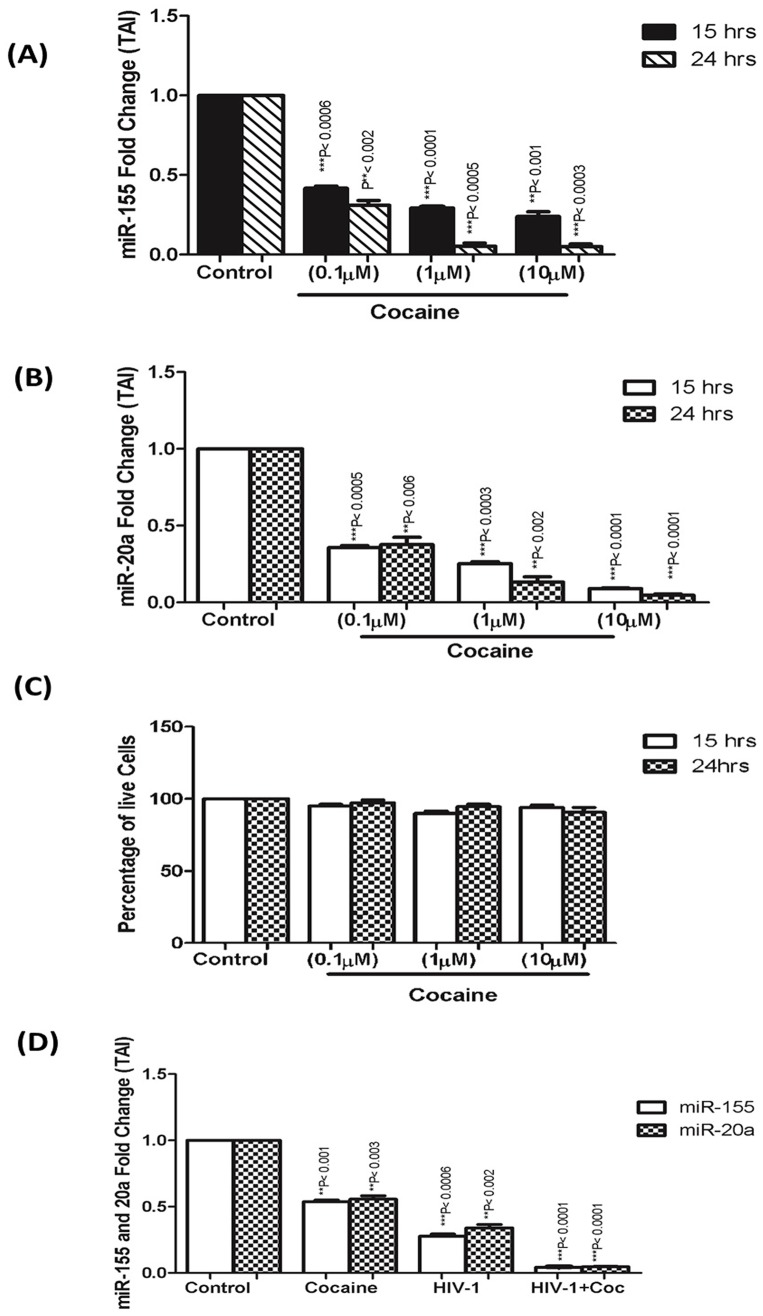
Cocaine and HIV synergistically down-regulate miR-155 and 20a. MDDCs (1×10^6^ cells/ml) from normal donors were cultured with 3 different concentrations (0.1, 1 and 10 µM) of cocaine for 15 and 24 hrs.Total cellular microRNA was extracted and amplified by specific RT-PCR for miR-155 and 20a gene expression. Cocaine down regulates **(A)** miR-155 **(B)** and 20a gene expression. (**C)** Cocaine treatment does not induce cytotoxicity. Cell suspension (200 µl) was subjected to XTT-PMS (100 µl) followed by a 4 hr incubation period. Cytotoxicity was measured by ELISA reader and cell suppression was calculated by: suppression index (%)  =  ((Abs cocaine – Abs blank)/(Abs control - Abs blank))×100%. (**D**) MDDCs (1×10 cells/ml) from normal donors were infected with HIV-1_BAL_ at a concentration of 20 ng for 2 hr, followed by treatment ± cocaine (1 µM) for 7 days. Total cellular microRNA was extracted and amplified by specific RT-PCR for miR-155 and 20a gene expression. Results shown are representative of mean ± SEM of three experiments. Graphs are represented as % of TAI compared to the non-treated control. p-value≤0.05 were considered to be significant.

Cocaine is known to increase the risk of acquiring HIV-infection and exacerbate the progression of HIV-associated neurological disorders [34]–[36]. MiR-155, a crucial molecule in the regulation of immune cell functions, [37] is shown to be increased during DC maturation and suppressed by HIV [38]. miR-20a is also reported to be suppressed by HIV [10]. Since cocaine has been shown to impact miR expression, we examined the effect of cocaine on miR-155 and -20a. MDDC (1×10^6^ cells/ml) were cultured for 15 and 24 hrs with different concentrations (0.1 µM -10 µM) of cocaine. Cocaine significantly down-regulated the expression of miR-155 in a dose-dependent manner at 15 hr (Fig. 1A). At 24 hr, cocaine produced even greater suppression of miR-155 gene expression, compared to 15 hr treatment (Fig. 1A). Similar to miR-155, expression of miR-20a was also significantly inhibited by cocaine in a dose dependent fashion at 15 hr, compared to untreated control (Fig. 1B). At 24 hr, cocaine produced greater suppression of miR-20a, similar to miR-155 (Fig. 1B). Based on our observations, a cocaine concentration of 1 µM was used in subsequent studies.

### Cocaine Treatment Does Not Induce Cytotoxicity

To ensure that observed down-regulation was not due to non-specific toxicity induced by cocaine, MDDCs were cultured with cocaine at different concentrations (0.1 µM, 1 µM, 10 µM), and cell cytotoxicity was measured by XTT assay as described [33]. Cocaine at 0.1–10 µM did not cause any toxicity, compared to controls (Fig. 1C).

### Cocaine and HIV-1 synergistically suppress miR-155 and miR-20a

**Figure 2:**
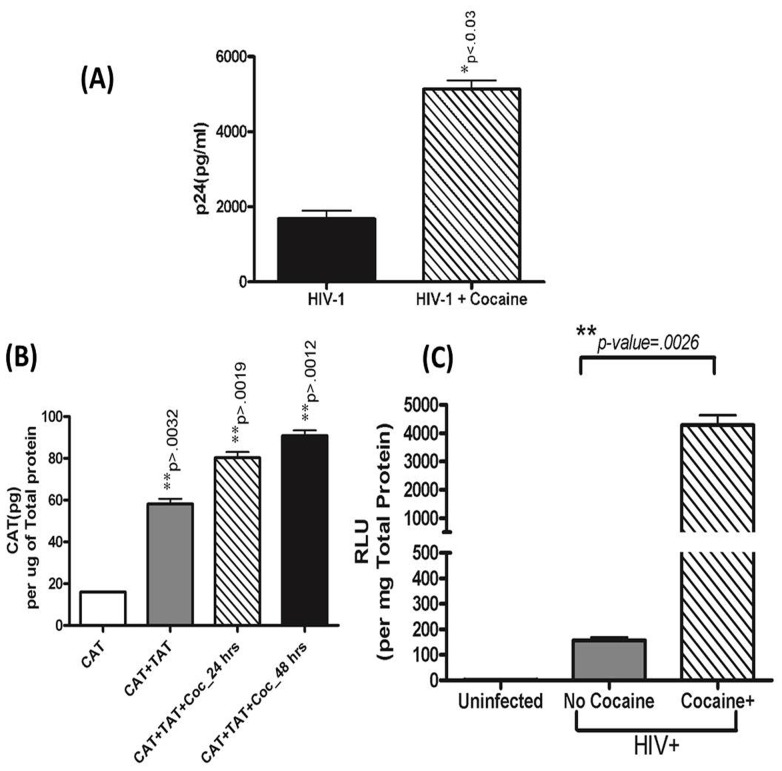
HIV replication increased in MDDCs treated with cocaine. **(A)** MDDCs (1×10^6^ cells/ml) from normal donors were infected with HIV-1_BAL_ at a concentration of 20 ng for 2 hr, followed by treatment ± cocaine (1 µM) for 7 days. The culture supernatants were quantitated for p24 Ag. Significance is calculated with respect to HIV-1 treated control cells. (**B**) Effect of cocaine on HIV-1 transcription was determined using an LTR-CAT reporter data normalized to total protein concentration. (**C**) HIV-1 transcription was determined in MDDCs from normal donors infected with HIV-luciferase viruses competent for a single round of infection (∼20 ng per (1×10^6^ cells) in the presence/absence of cocaine (1 µM) for 72 hours. Cultures were harvested 72 hours post infection and luciferase assayed. Results shown are representative of mean ± SEM of three experiments. p-value≤0.05 were considered to be significant.

Previous studies have shown that HIV-1 suppresses certain miRNAs during virus replication [10]. We investigated whether cocaine and HIV-1 synergize to modulate miR-155 and 20a. MDDC were infected with HIV-1(20 ng) and treated with/without cocaine for 4 days. HIV-1 significantly suppressed both miRNAs compared to untreated control (Fig. 1D), and HIV-1 in combination with cocaine showed a synergistic effect on miRNA expression. We examined whether the cocaine-induced down-regulation of miR-155 and -20a is associated with concomitant up-regulation of HIV transcription and p24 production. We found, as measured by p24 ELISA, an almost 3-fold up-regulation in HIV production with cocaine exposure (Fig. 2A), suggesting that cocaine may significantly impact HIV transcription. To verify cocaine-mediated effects on transcriptional activation, we used a HIV-LTR-CAT reporter. Cells were transfected with pSVTat and pHIV-LTR-CAT, treated with cocaine for 48 hrs (24 hr post the initial LTR-CAT transfection), harvested after three days post transfection, and assessed for CAT concentration. We observed that cocaine increased HIV transcription by 5-fold (Fig. 2B). To further verify long-term effects of cocaine on HIV transcription, we infected MDDCs with a HIV-luciferase pseudo virus (capable of only single-round replication) in the presence or absence of cocaine (Fig. 2C). Viral transcription increased almost 30-fold in cocaine-treated MDDCs, compared to the infected MDDCs not exposed to cocaine. ‘The observed cocaine-mediated effect on HIV replication and transcription could also in part be due to increased infectivity of the pseudotyped HIV-luciferase virus.’ Together, these results suggested that cocaine exposure enhanced HIV-1 transcription and that increased viral production in cocaine-treated MDDCs may be associated with the down-regulation of miR-155 and 20a.

### Increased miR-155 expression Inhibits HIV-1 Replication

**Figure 3:**
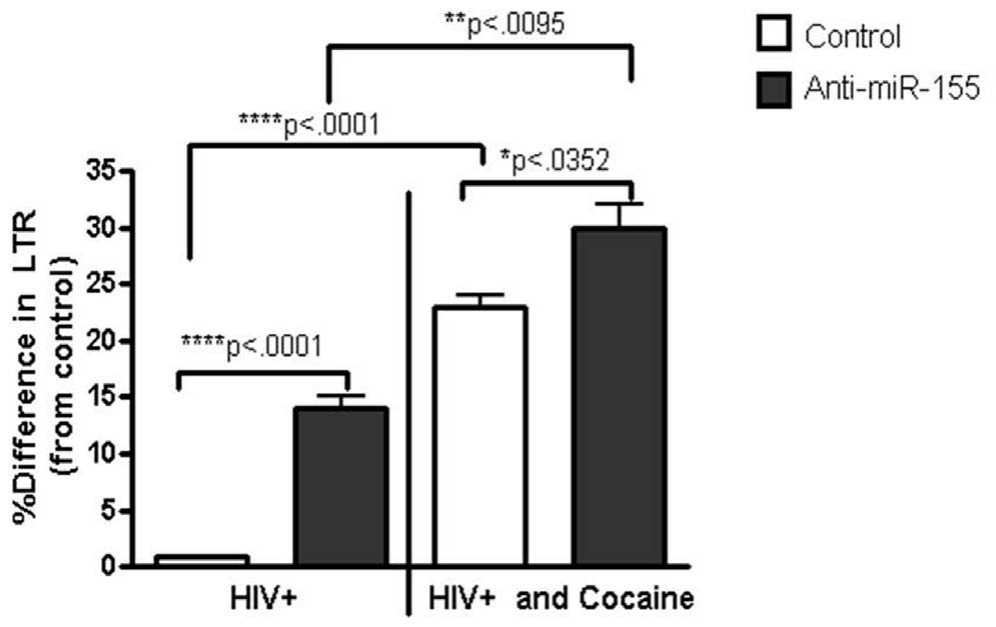
miR-155 inhibition enhances HIV-1 replication. MDDCs (1×10^6^ cells/ml) from normal donors were transfected with anti-miR-155, at a final concentration of 75 nM, followed by infection with HIV-1 (20 ng) and treatment ± cocaine (1 µM). Total RNA was extracted and amplified by RT-PCR for HIV-1 LTR/RU-5 gene, to detect early steps in HIV reverse transcription. Significance is calculated with respect to non targeting HIV-1 control cells. Graphs are represented as % difference from control. Results shown are representative of mean ± SEM of three experiments. p-value≤0.05 were considered to be significant.

To further investigate the exact role(s) of miR-155 on cocaine mediated modulation of HIV-1 replication, we transfected MDDCs with either miR-155 mimic or anti-miR-155, followed by infection with HIV-1 and/or cocaine treatment. Total RNA was extracted and amplified by qRT-PCR for HIV-1 LTR/R-U5 [31], [39], [40]. miR-155 over expression significantly suppressed HIV-1 replication whereas anti-miR-155 transfection in combination with HIV-1 infection enhanced HIV-1 replication compared to non-transfected HIV-1 control (Fig. 3). MDDCs transfected with miR-155 mimic and infected with HIV-1 in the presence of cocaine significantly down-regulated HIV-LTR-R/U5 (Data not shown), whereas MDDCs transfected with anti-miR-155 and infected with HIV-1 in the presence of cocaine significantly up-regulated HIV-LTR-R/U5, compared to cocaine-treated cells transfected with the negative control (Fig. 3). These results suggest that MDDCs become more susceptible to HIV infection, when miR-155 is inhibited.

### Over-expression miR-155 down-regulates PU.1

**Figure 4:**
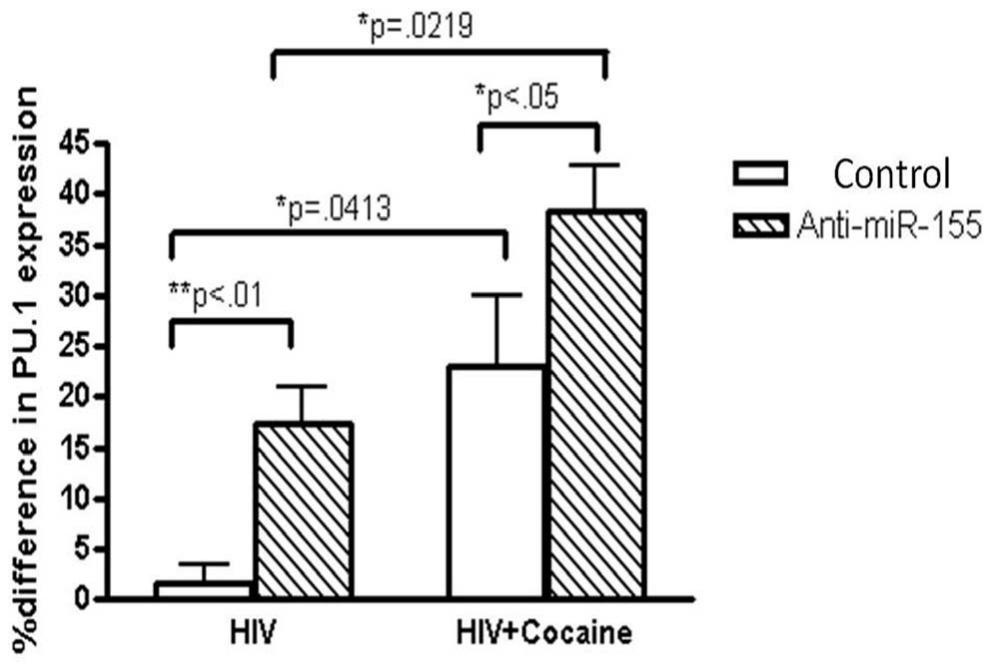
miR-155 modulates PU.1 levels. MDDCs (1×10^6^ cells/ml) from normal donors were transfected with anti-miR-155, at a final concentration of 75 nM, followed by infection with HIV-1 (20 ng) and treatment ± cocaine (1 µM). Total RNA was extracted and amplified by RT-PCR for PU.1 gene expression. Values were normalized against GAPDH and significance is calculated with respect to control. Graphs are represented as % difference from control. Results shown are representative of mean ± SEM of three experiments. p-value≤0.05 were considered to be significant.

PU.1 has been reported to be a direct transcription factor of miR-155, and has been indirectly linked to regulate DC-SIGN expression [14], [21]. To better understand the mechanism of cocaine-induced modulation of HIV replication, we investigated the effects of miR-155 on the regulation of PU.1, by transfection experiments. Over-expression of miR-155 suppressed PU.1 expression, while blocking miR-155 expression enhanced PU.1 expression, compared to non-targeting control (Fig 4). MDDCs transfected with miR-155 mimic and treated with cocaine decreased PU.1 expression whereas anti-miR-155 transfection and subsequent cocaine treatment increased PU.1 expression, compared to non-targeting cocaine control (Fig. 4). Over-expression of miR-155 followed by HIV-1 infection showed a down-regulation in PU.1 expression whereas transfection with anti-miR-155 showed an up-regulation, compared to non-targeting HIV-1 control. Decreased PU.1 expression was observed with miR-155 over-expression, followed by HIV-1 infection and cocaine treatment, whereas an up-regulation was seen with anti-miR-155 transfection, compared to respective non-targeting control. Non-targeting cocaine control increased PU.1 expression, compared to negative control. PU.1 expression in the HIV-infected control is decreased almost 28% when compared to the uninfected control. Interestingly, cocaine increased PU.1 in control miRNA while HIV infection decreased PU.1. We observed no significant difference in PU.1 expression from uninfected control.

### miR-155Cocaine enhances miR-155 mediated DC-SIGN upregulation

**Figure 5:**
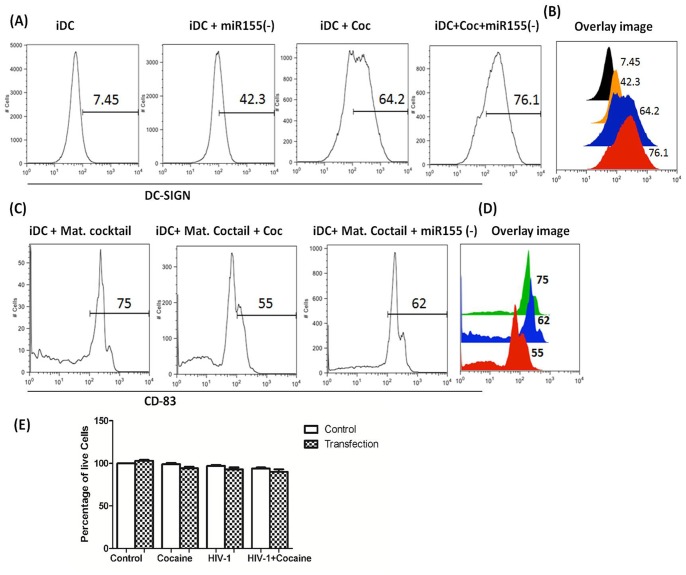
Cocaine increases DC-SIGN expression and prevents DC maturation by down regulating miR-155. (**A**–**B**) MDDCs at Day 6 were transfected with anti-miR-155 or anti-miR-Control, at a final concentration of 75 nM, followed by treatment with or without cocaine (1 µM) for 3 days. Non-transfected, untreated cultures served as controls. Cells were stained with antibody against DC-SIGN and the membrane expression was assessed by flow cytometry and presented as graphs showing percentage of positive population (%). (**C**–**D**) Following transfection and treatment with or without cocaine, LPS (100 ng/mL; for DC maturation) was added to all wells and left for 2 days. At the end of treatment, cells were stained with antibody against CD83 (DC maturation marker) and was assessed by flow cytometry. Representative graphs show percentage of positive population (%). (**E**) Over-expression of miR-155 does not cause cytotoxicity in MDDC. Cell suspension (200 µl) was subjected to XTT-PMS (100 µl) followed by a 4 hr incubation period. Cytotoxicity was measured by ELISA reader and cell suppression was calculated by: suppression index (%)  =  (Abs cocaine – Abs blank)/(Abs control - Abs blank))×100%.

DC-SIGN facilitates HIV-1 infection independent of CD4 and/or co-receptors [16]–[18]. Previous studies have shown miR-155 expression down-regulated DC-SIGN expression [21]. Our lab has reported that cocaine up-regulates DC-SIGN expression on MDDCs [31]. We hypothesize that cocaine regulates DC-SIGN expression through miR-155 down regulation. To explore this hypothesis we examined the effect of cocaine on DC-SIGN expression. MDDCs were transfected with a miR-155 inhibitor in the presence/absence of cocaine and DC-SIGN expression was monitored by flow cytometry. Results show that cocaine increased the percentage of DC-SIGN positive cells by 2-fold compared to untreated cells (Fig. 5A). This is consistent with previous findings [31]. Similar to cocaine-treatment, miR-155 inhibition also increased DC-SIGN, whereas the negative control miRNA did not have any effect. Moreover, miR-155 inhibition, in the presence of cocaine significantly enhanced DC-SIGN (Fig 5A–B). These findings suggest that cocaine mediated up-regulation of DC-SIGN is through suppression of miR-155.

### Cocaine impairs maturation of MDDCs

miR-155 has been reported to be up-regulated during DC maturation. Given the role of miR-155 in DC maturation, we examined the impact of cocaine on DC maturation. Since our previous experiments showed that cocaine down-regulated miR-155, we wanted to determine whether DC maturation was impaired by cocaine. To test this, MDDCs were transfected with either a miR-155 inhibitor or a negative control miRNA. Maturation of MDDCs was induced by adding LPS to the culture media in the presence or absence of cocaine. Following a 2 day exposure, we examined the expression of CD83, a DC maturation marker. An increase in CD83 expression was observed upon DC maturation in the absence of cocaine (Fig. 5C–D). However, in the presence of cocaine CD83 expression was significantly reduced. Furthermore, cells transfected with the miR-155 inhibitor also exhibit reduced CD83 expression. No difference in CD83 expression was observed in cells transfected with the negative control miRNA when compared to the untransfected control (data not shown). Taken together, these results indicate that cocaine impairs DC maturation through the down regulation of miR-155 expression. To rule out the possibility that transient transfection could activate apoptosis or cause direct cell death we measured cytotoxicity in MDDCs that were transfected with mimic miR-155 and infected with HIV-1 with/without cocaine treatment. The results indicated that over-expression of miR-155 does not cause cytotoxicity in MDDCs (Fig 5E), thus validating the observation of cocaine induced modifications on miR-155 expression.

## Discussion and Conclusion

Several investigators have previously demonstrated that cocaine promotes HIV pathogenesis [4], [31], [41], [42]. In HIV infection as well as in other infections, MDDCs are the first line of defense and have been suggested to be the initial target of infection [4], [31], [43], [44]. During maturation of human MDDCs, levels of miR-155 increase [21], [38] and once DCs become mature they are less susceptible to HIV-1 infection [31]. Interestingly, miR-155 has been associated with increased susceptibility to HIV-1 infection [21]. The polycistronic miR-17/92 cluster, which encodes miR-20a, is believed to play a physiological role in controlling HIV-1 replication [10]. We hypothesized that cocaine may affect miR-155 levels and thus indirectly increase HIV susceptibility in MDDCs. In this study, we examined the *in vitro* effect of cocaine on miR-155 and 20a expression, and subsequent impact on HIV transcription. We demonstrated that cocaine significantly reduced, in a dose dependent manner, miR-155 and miR-20a genes in MDDCs. Our results show that even the highest concentration of cocaine used (10 µM) was not cytotoxic to MDDCs, thus ruling out the possibility of non-specific down-regulation of miRNAs.

It has been previously reported that DC-SIGN is reduced when cells overexpress miR-155 [14], [21], [38] and that cocaine facilitates neuropathogenesis of HIV-1 infection [4]. Our results suggest that cocaine suppresses miR-155 expression; thereby, increasing DC-SIGN expression on MDDCs, possibly preventing the maturation of dendritic cells, and increasing susceptibility to HIV-1 infection. This is the first report demonstrating that cocaine suppresses miR-155 and miR20a. We show that HIV, similar to cocaine, suppressed the miR-155 and miR-20a expression compared to the uninfected control. Together HIV and cocaine synergistically lowered miR-155 and miR-20a compared to HIV-1 or cocaine alone. Consistent with previous studies [4] we show that cocaine significantly enhances HIV-1 infection in MDDCs. Overexpression of miR-155 in MDDCs demonstrated reduced levels of HIV LTR/R-U5 while inhibition of miR-155 enhanced LTR/R-U5 gene expression. This indicates that the MDDCs have become more permissive to HIV infection in the absence of miR-155, suggesting a role for miR-155 in HIV transcription. The presence of cocaine further lowered miR-155 expression and exacerbated the observed effect. Taken together, these results suggest that a miR-155 mimic has the potential to reduce HIV-1 replication and raises the possibility of targeting miR-155 as a novel therapeutic in HIV-1 infected cocaine abusers.

Reduction of PU.1, one of the direct functional targets for miR-155 [21], [22], [45], is associated with lowered DC-SIGN expression during the maturation of MDDCs.[24]. Based on these previous studies, we investigated the role of cocaine on miR-155 regulation of PU.1 expression. We also found that cocaine up-regulated PU.1 expression and that this is mediated by the down-regulation of miR-155. This effect may be attributed to cocaine-mediated enhancement of HIV-1 pathogenesis, since increased PU.1 is linked to elevated DC-SIGN [21]. We observed that HIV-1 decreased PU.1 levels rather than an increase in the levels, as expected. This observation may be due to the fact that in HIV infection multiple miRNAs may be differentially expressed thereby limiting the overall effect on PU.1 expression. Previous studies show that PU.1-deficient mice exhibit defective DC development [46], [47] suggesting that PU.1 is integral for DC development. PU.1 is likely to have multiple targets within DCs since it has already been shown to regulate a large number of myeloid genes [48], [49]; including DC-SIGN in myeloid cells [21].

Our study and previous reports suggest that miR-155 regulates the DC-SIGN expression at the transcriptional level, via directly targeting PU.1. The role of DC-SIGN in HIV infection and regulation of DC-SIGN by miR-155 prompted us to examine the role of miR-155 in HIV transcription and DC-SIGN expression. A decrease in DC-SIGN was reported with the over-expression of miR-155 [21]. Here, we show an increase in DC-SIGN with miR-155 inhibition. Cocaine treatment also increased DC-SIGN, which is in-line with our previous report. Further, miR-155 and cocaine showed a synergistic effect on DC-SIGN expression. This suggests that, increased DC-SIGN expression by cocaine is mediated through miR-155 modulation.

During maturation of human MDDCs, levels of miR-155 increase [5], [14], [21] and DC-SIGN expression is lowered [50], which is associated with a reduction of PU.1. Although there have been conflicting studies, increased miR-155 upon TLR stimulation is shown to up-regulate CD86, CD40, and IL12p40 [51]. In this study, we found a reduction in the number of cells expressing CD83 with miR-155 inhibition indicating reduced MDDCs maturation. Cocaine also reduced CD83 on MDDCs. As DCs become mature they are less susceptible to HIV-1 infection. This suggests that cocaine-mediated suppression of miR-155 could prevent the maturation of dendritic cells resulting in increased DC-SIGN expression and susceptibility to HIV-1 infection. Prevention of maturation may lead to poor antigen presentation, poor immunogenicity, and increased HIV immune evasion.

**Figure 6:**
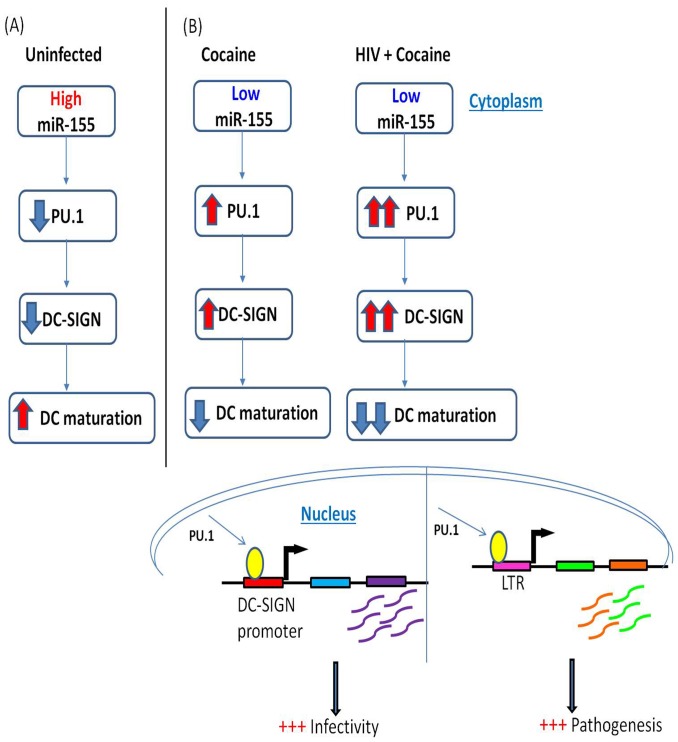
Overview of Cocaine-enhancement of HIV infectivity in MDC via the down-regulation of miR-155. (**A**) In absence of cocaine or HIV miR155 targets DC-SIGN and PU.1, reducing the expression post-transcriptionally. Lowered PU.1 and DC-SIGN expression is observed in mature DCs. (**B**) In the presence of Cocaine or HIV alone miR-155 levels are reduced resulting in elevated PU.1 and DC-SIGN expression. This phenotype is evident in immature DCs (iDCs). Together HIV and cocaine, synergize to dramatically lower miR-155 resulting in elevated levels of the transcription factor Pu.1. In turn, Pu.1 binds sites within the DC-SIGN promoter as well as the HIV LTR and promotes transcription. The resulting elevated DC-SIGN expression in DCs lead to maintenance an immature DC phenotype and thus increased HIV infectivity. By binding NF-κB sites within the LTR, PU.1 increases viral transcription and pathogenesis.

In summary, we show for the first time that cocaine down-regulates cellular miR-155 and 20a. Figure 6 summarizes an overview of cocaine mediated enhancement of HIV infectivity in MDC via the down-regulation of miR-155. Given the impact of miR-155 in DC-SIGN expression and the role of miR-155 in DC maturation, the down-regulation of miR-155 by cocaine suggest a role for cocaine-mediated enhancement of HIV permissiveness in MDDCs. Our results suggest that the mechanism of cocaine mediated enhancement of HIV infection may be mediated by the down-regulation of miR-155 and this could lead to faster progression of HIV disease. Overall, results from our study could potentially lead to the development of novel miRNA-based therapeutic strategies targeting cocaine addiction and HIV infection.
